# Coral-Derived Natural Marine Compound GB9 Impairs Vascular Development in Zebrafish

**DOI:** 10.3390/ijms18081696

**Published:** 2017-08-03

**Authors:** Yi-Chun Song, Bao-Jueng Wu, Chien-Chih Chiu, Chun-Lin Chen, Jun-Qing Zhou, Shuo-Rong Liang, Chang-Yih Duh, Ping-Jyun Sung, Zhi-Hong Wen, Chang-Yi Wu

**Affiliations:** 1 Department of Biological Sciences, National Sun Yat-sen University, Kaohsiung 80424, Taiwan; spk91326@gmail.com (Y.-C.S.); cchiu@kmu.edu.tw (C.-C.C.); chunlinchen@mail.nsysu.edu.tw (C.-L.C.); angeldie830801@gmail.com (J.-Q.Z.); curiousbaby0911@gmail.com (S.-R.L.); 2 Department of Marine Biotechnology and Resources, National Sun Yat-sen University, Kaohsiung 80424, Taiwan; bununabo@gmail.com (B.-J.W.); yihduh@mail.nsysu.edu.tw (C.-Y.D.); wzh@mail.nsysu.edu.tw (Z.-H.W.); 3 Zuoying Branch of Kaohsiung Armed Forces General Hospital, Kaohsiung 81342, Taiwan; 4 Department of Biotechnology, Kaohsiung Medical University, Kaohsiung 80708, Taiwan; 5 Research Center for Environment Medicine, Kaohsiung Medical University, Kaohsiung 80708, Taiwan; 6 National Museum of Marine Biology and Aquarium, Pingtung 94450, Taiwan; pjsung@nmmba.gov.tw; 7 Doctoral Degree Program in Marine Biotechnology, National Sun Yat-sen University, Academia Sinica, Kaohsiung 80424, Taiwan

**Keywords:** marine compound GB9, intersegmental vessel, vascular development, zebrafish, oxidative stress

## Abstract

Blood vessels in vertebrates are established and genetically controlled in an evolutionarily-conserved manner during embryogenesis. Disruption of vascular growth by chemical compounds or environmental hormones may cause developmental defects. This study analyzed the vascular impacts of marine compound GB9 in zebrafish. GB9 was isolated from the marine soft coral *Capnella imbricata* and had shown anti-neuroinflammatory and anti-nociceptive activities. However, the role of GB9 on vascular development has not been reported. We first tested the survival rate of embryos under exogenous 5, 7.5, 10, and 15 μM GB9 added to the medium and determined a sub-lethal dosage of 10 μM GB9 for further assay. Using transgenic *Tg*(*fli:eGFP*) fish to examine vascular development, we found that GB9 treatment impaired intersegmental vessel (ISV) growth and caudal vein plexus (CVP) patterning at 25 hours post-fertilization (hpf) and 30 hpf. GB9 exposure caused pericardial edema and impaired circulation at 48–52 hpf, which are common secondary effects of vascular defects and suggest the effects of GB9 on vascular development. Apoptic cell death analysis showed that vascular defects were not caused by cell death, but were likely due to the inhibition of migration and/or proliferation by examining ISV cell numbers. To test the molecular mechanisms of vascular defects in GB9-treated embryos, we examined the expression of vascular markers and found the decreased expression of vascular specific markers *ephrinb2*, *flk*, *mrc1*, and *stabilin*. In addition, we examined whether GB9 treatment impairs vascular growth due to an imbalance of redox homeostasis. We found an enhanced effect of vascular defects during GB9 and H_2_O_2_ co-treatment. Moreover, exogenous *N*-acetyl-cysteine (NAC) treatment rescued the vascular defects in GB9 treated embryos. Our results showed that GB9 exposure causes vascular defects likely mediated by the imbalance of redox homeostasis.

## 1. Introduction

**Figure 1:**
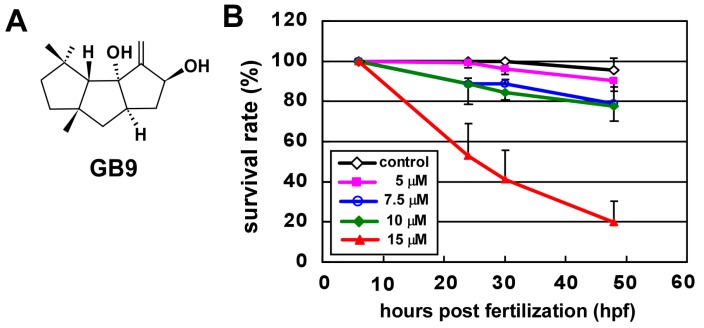
Chemical structures of capnellene (GB9) and toxic effects of GB9 on zebrafish. (**A**) Capnellene (GB9: 4,4,6a-trimethyl-3-methylene-decahydro-cyclopenta[ə]pentalene-2,3a-diol) has a molecular weight of 204 g/mol; (**B**) Wild-type zebrafish embryos (*n* = 75, 80, 50, and 68) were treated with various concentrations of GB9 (0, 5, 7.5, 10, and 15 μM) at 6 hours post-fertilization (hpf), with survival rates recorded at 24, 30, and 48 hpf. The survival ratio at 6 hpf is set as 100%.

Natural compounds discovered in marine organisms have drawn attention as potential sources of novel drugs for human diseases [1]. Many studies have indicated that marine natural products exhibit anti-inflammatory bioactivities that can be used to develop antiviral and anti-inflammatory drugs [2,3,4,5]. Such products also have the potential to inhibit angiogenesis and prevent cardiovascular disease or can be used to develop anti-tumor drugs [6,7]. Capnellene was first isolated from the soft coral *Capnella imbricata* in Indonesia [8]. One of the compounds GB9, Δ^9(12)^-capnellene-8β, 10α-diol (4,4,6a-trimethyl-3-methylene-decahydro-cyclopenta[ə]pentalene-2,3a- diol (Figure 1A), used in this study was first isolated from the same species of soft coral in Taiwan [9]. At a concentration of 10 µM in macrophage cells, GB9 suppresses the lipopolysaccharide-induced expression of the pro-inflammatory proteins, inducible nitric oxide synthase (iNOS) and cyclooxygenase-2 (COX-2), indicating the anti-inflammatory activity of GB9 [10]. In addition, GB9 has been reported to have an anti-neuroinflammatory function in Interferon-γ (IFN-γ)-stimulated microglial cells. Inflammation is a pathophysiological state usually associated with pain. Jean et al. [10] further showed, in rats, that GB9 reduced chronic constriction injury-induced neuropathic pain. Therefore, capnellene may serve as a useful compound to develop novel therapeutic drug for inflammatory-related diseases. However, the effect of GB9 on the vasculature is not known, nor are the potential side effects it may produce when used to treat inflammatory-related diseases. In addition, anti-angiogentic activity can be used to treat related vascular diseases.

The establishment and precise patterning and regulation of blood vessels in vertebrates is required for embryonic survival [11]. Vasculogenesis and angiogenesis are the main processes by which blood vessels are formed. During vasculogenesis, the arteries and veins are de novo formed from the migration and differentiation of angioblast progenitors [12,13]. After the establishment of these vessels, new blood vessels sprout from existing vessels and develop into mature vessels through a process called angiogenesis [14]. The zebrafish is an ideal organism through which to explore vascular development because of the optical transparency of embryos and the availability of effective genetic tools [15]. In addition, the cellular and molecular mechanisms underlying vascular development have been shown to be conserved in vertebrates. The zebrafish model has been successfully used to reveal molecular mechanisms of blood vessel formation, such as angioblast specification and differentiation, endothelial cell proliferation and migration, vessel formation, patterning, and morphogenesis [16,17,18,19,20,21,22,23]. In addition, the zebrafish model has been widely used to examine the effects of chemicals, drugs, and environmental hormones in vascular development [24,25].

The formation of arteries and veins is specified from angioblast progenitors mainly controlled via vascular endothelial growth factor (VEGF)-Notch signaling pathways [12,20]. After the main axial vessels have formed, angioblasts undergo further proliferation and migration to form a patterned network of smaller vessels. The early, rapid growth of stereotypic intersegmental vessels (ISVs) in the trunks of zebrafish makes them ideal for investigating angiogenesis in vivo. Many genetic molecules have been identified as mediating the timing and guidance of sprouting in these vessels. In addition to sprouting dorsally to form ISVs, recent studies have shown that endothelial cells in the tail region also sprout ventrally from axial veins to form a honeycomb-like structure called the caudal vein plexus (CVP) via distinct BMP signal pathways [26,27]. The understanding of molecular mechanisms and signals controlling vascular growth in zebrafish provides an excellent platform to test the impact of chemicals or drugs on vascular development.

Oxidative stress is defined as an imbalance between reactive oxygen species (ROS) and antioxidant systems. ROS, including the superoxide anion, hydrogen peroxide (H_2_O_2_), and the hydroxyl radical can cause various types of biological damage and are implicated in the pathogenesis of vascular dysfunction and atherogenesis [28,29]. In addition, treatment using chemicals or natural products can result in anti-proliferation and migration of endothelial cells or the death of cancer cells mediated by the increase of oxidative stress [30,31]. Interestingly, it has been shown that lower ROS levels can act as an intracellular signal, known as “redox signaling”, to mediate normal physiological vasculature processes [32]. Thus, optimal ROS signals are known to be involved in many cellular functions, including proliferation, migration, and differentiation. ROS are commonly generated through normal aerobic metabolism in the mitochondria, or through exogenous exposure to stress conditions, such as radiation, heat shock, and chemical treatment [33]. Genetic disruption of antioxidant genes, such as *Grx2* or *Prdx1*, results in vascular defects mediated by the imbalance of redox homeostasis [34,35]. Some marine products have been reported to have anticancer effects mediated by ROS generation [6,36].

This study used zebrafish to analyze the vascular impacts of marine compound GB9. We demonstrated a reduction of both ISV cells and CVP endothelial cells sprouting, and decreased loop formation in GB9-treated embryos, indicating impairment of cell proliferation and migration. We found that GB9 treatment results in decreased expression of vascular markers and caused vascular defects which are likely mediated by the increase of oxidative stress. Our results provide a useful reference for future examination of vascular growth during chemical treatment.

## 2. Results

### 2.1. GB9 Treatment Causes Vascular Defects during Zebrafish Development

We are interested in characterizing genes that control vascular development and identifying molecules that have pro- or anti-angiogenetic attributes. We analyze the effects of GB9 (Figure 1A) on vascular function in zebrafish, beginning with toxicity on zebrafish embryos. We found that a 15 μM GB9 treatment causes a high rate of mortality (~80%) with malformed morphology at 48 h post-fertilization (hpf). However, at a concentration of 5–10 μM, embryos showed survival rates of over 80% without malformation in fish shape (Figure 1B). Even five days post-fertilization (dpf), there was a ~60% survival rate among embryos treated with 10 μM GB9 (Figure S1). Since the main vascular development and patterning occurred within 4 dpf in zebrafish, we used 10 μM GB9 for treatment in further experiments.

**Figure 2:**
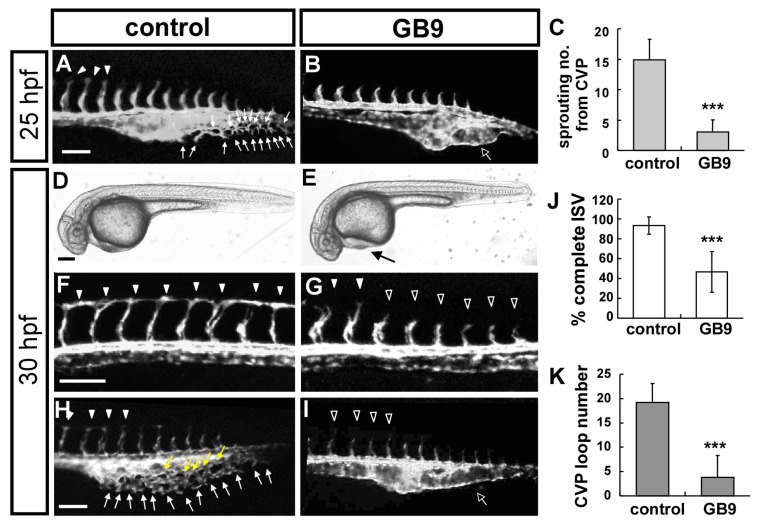
GB9 treatment causes vascular defects during zebrafish development. (**A**,**B**) Lateral view of GB9 treatment shows less ISV growth at the top of embryo and less or no angiogenic sprouting from the caudal vein plexus (CVP) (**hollow** **arrow** in (**B**)) compared to untreated controls (**arrows** in (**A**)) in *Tg*(*flk:eGFP*) that expressed GFP in endothelial cells. (**C**) Quantification of endothelial sprouting at CVP shows a five-fold decrease in GB9-treated fish (*n* = 12 in control and *n* = 12 in GB9 sample) at 25 hpf. (**D**–**H**) At 30 hpf, bright field images showed GB9 treatment causes minor pericardial edema ((**E**), **arrow**). In untreated control embryos, ISVs reach the DLAV at the embryo’s dorsal aspect (**arrowheads** in (**F**,**H**)) and the CVP formed loop structures at the tail ((**H**), **arrows**). At the same stage, ISVs show slower growth at mid-somite in the GB9 treated embryos (**hollow arrowheads** in (**G**,**I**)) and less endothelial cell sprouting and loop formation at CVP (**hollow** **arrow** in (**I**)). (**J**,**K**) Quantification of percentage of completed ISV and CVP structures in GB9-treated fish at 30 hpf shows a ~2.2-fold and ca. six-fold reduction, respectively (*n* = 12 in control and *n* = 15 in GB9 treated samples). *** refers to *p* < 0.0001 by an unpaired Student’s *t*-test. Scale bars are 100 μm for (**A**,**B**,**F**–**I**), and 200 μm for (**D**,**E**).

With 10 μM GB9 treatment in transgenic *Tg*(*flk:eGFP*), at 25 hpf, GB9-treated embryos showed little or no angiogenic sprouting from the caudal vein compared to untreated controls (Figure 2A–D) with a five-times decrease (*n* = 12 in control and GB9-treated embryos) (Figure 2C,D,K). At 30 hpf, we observed a slight pericardial edema during zebrafish development (Figure 2E,F). Meanwhile, in untreated control embryos, ISVs reached the dorsal longitudinal anastomotic vessel (DLAV) at the dorsal region of the embryo and the CVP formed loop structures at the tail. At the same stage, ISVs were stalled at mid-somite in the GB9 treatment group with a ~45% incomplete ISV structure and ca. five-fold decrease in CVP loop structures at the tail region compared to controls (*n* = 12 in control and *n* = 15 in GB9-treated embryos) (Figure 2G–J,L,M). The results suggest GB9 treatment inhibits vascular growth during zebrafish development.

We carefully examined the impact of GB9-treatment on whole embryos starting from 6 hpf. No obvious defects were observed during the development. At 24–25 hpf, we assessed the gross developmental process in GB9-treated embryos and found that the expression of *myoD* (somite marker), *Hbbe1* (blood marker), and *sox3* and *shh* (neural markers) were unaffected, suggesting that GB9-treatment did not affect the development of somites, blood, or nerves (Figure S2A–H). Interestingly, the reduced expression of *cmlc2* (heart marker), suggested GB9 also has an effect on heart development (Figure S2I,J,I’,J’). The reduced heart rate was consistent with defects in heart function found in GB9-treated embryos (Figure S2K). However, we observed no significant developmental delay in GB9-treated embryos by measuring the number of somites from 12 to 21 somite stages (~15–20 hpf) and counting the number of embryos with heart beat at 24–25 hpf (data not shown). At 48 hpf, body and eye size measurements found no difference in body size, but a smaller eye diameter (Figure S3), suggesting that GB9 has an effect on vascular, heart and eye development, however, the most significant impact is on the vasculature.

### 2.2. GB9 Treatment Results in Edema and Circulation Defect

**Figure 3:**
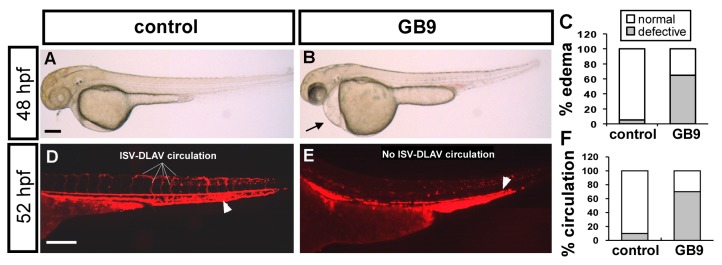
Pericardial edema and circulation defect in GB9 treated embryo. (**A**–**C**) At 48 hpf, representative edema fish (**arrow** in (**B**)) and quantitative results showed 60% of GB9 treated embryo (*n* = 20) with mild to severe pericardial edema compared to untreated control (*n* = 19). (**D**,**E**) GB9 treatment in transgenic *Tg*(*gata1:dsRed*) embryos with dsRed-labeled blood cells showed slow to no circulation at 52 hpf (**E**) compared to wild-type fish (**D**). (**F**) Circulation defects at the ISV/DLAV or slow to lose axial circulation of the aorta/vein in the trunk region are quantified in control (*n* = 18) and GB9 treated embryo (*n* = 18) at 52 hpf. The scale bar in (**A**,**B**) is 500 μm, and in (**D**,**E**) represents 200 μm.

We observed slight edema at earlier 30 hpf in GB9 treatment embryos. At 48–52 hpf, we found that GB9 treatment caused an increase in pericardial edema. At 48 hph, treated embryos showed ~70% pericardial edema, as opposed to 10% for controls (*n* = 19 in wt and *n* = 20 in GB9-treated embryos) (Figure 3A–C). Since edema and loss of circulation are common secondary effects of vascular malformation, we further examined the blood flow in wild-type and GB9-treated embryos using transgenic *Tg*(*gata1:dsRed*) embryos with dsRed-labeled blood cells. Among GB9-treated embryos, ~65% showed slow to no circulation in the axial vessels and/or in ISV-DLAV at 52 hpf compared to 92% for wild-type fish (*n* = 18 in control and *n* = 18 in GB9 treatment embryos) (Figure 3D–F). The results correspond to vasculature defects in the GB9 treatment fish.

### 2.3. Vascular Defects in GB9-Treated Embryos Are Likely Due to the Impairment of Proliferation and/or Migration of Endothelial Cells

**Figure 4:**
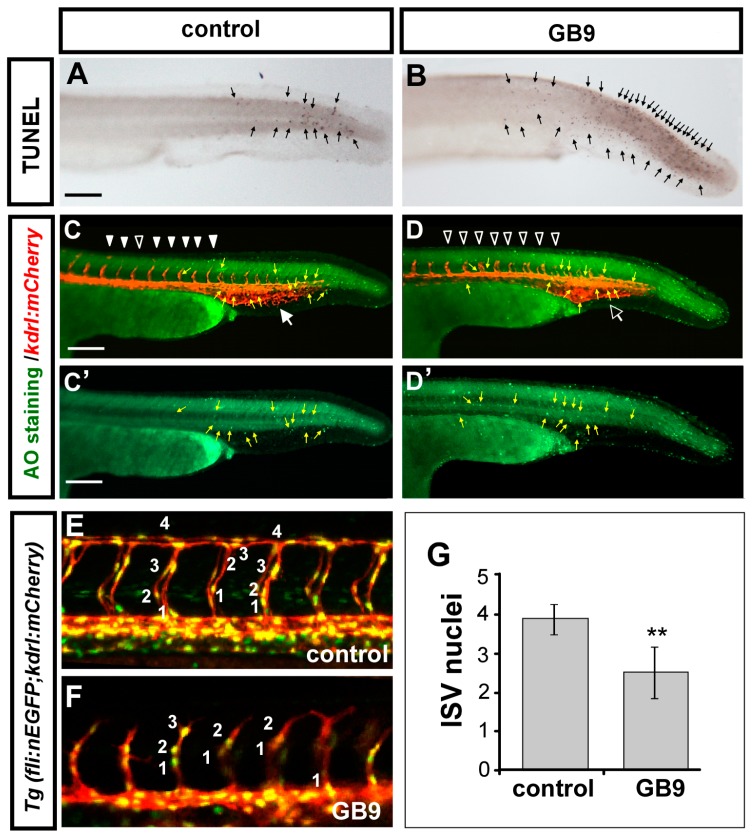
GB9 treatment inhibits the growth of ISV cells. (**A**–**D**) At 30 hpf, TUNEL labeling (in wild-type AB strain) and AO staining (in *Tg*(*kdrl:mCherry*) embryos) were used to detect apoptotic cells (**arrows** in (**A**,**B**) and green spots in (**C**,**C’**,**D**,**D’**)) in 0.3% DMSO control (**A**,**C**,**C’**), and GB9-treated embryo (**B**,**D**,**D’**). Some increased apoptotic cells were observed on the skin close to tail region in GB9 treated embryo, but not in vascular regions compared to untreated controls (**yellow arrows** in (**C**,**C’**,**D**,**D’**)). At 30 hpf, ISVs show slower growth at mid-somite in the GB9 treated *Tg*(*kdrl:mCherry*) embryos (**hollow arrowheads** in (**D**)) and less endothelial cell sprouting and loop formation at CVP (**hollow** **arrow** in **D**) compared to control embryos (**arrowheads** and **arrow** in (**C**). The number of cells forming each ISV counted in control *Tg*(*kdrl:mCherry; fli1a:negfp ^y7^*) (**E**) and GB9-treated embryos (**F**) at 30 hpf. (**G**) Quantification of average endothelial cells per ISV is 3.9 ± 0.3 (ISV *n* = 60) and 2.2 ± 0.5 (ISV *n* = 80) in control and GB9 treatment respectively. ** refers to *p* < 0.001 by an unpaired Student’s *t*-test. Scale bars are 100 μm for (**A**–**F**).

GB9 exposure resulted in vascular growth defects, suggesting the impairment of endothelial cellular proliferation and/or migration, or an increase in cell death in GB9-treated embryos. To test these hypotheses, we first performed terminal deoxynucleotidyl transferase dUTP nick end labeling (TUNEL) analysis and Acridine Orange (AO) assay to determine whether additional cell death occurred following GB9 treatment at 30 hpf. An increase in the number of apoptotic cells was observed on the skin close to the tail region in GB9-treated embryos (Figure 4A–D,C’,D’), however, no obvious increase of apoptotic cells was found in the vessels at the trunk and CVP regions in comparison to control embryos by using AO staining in *Tg*(*kdrl:mCherry*) embryos (Figure 4C,D,C’,D’), suggesting vascular defects were not due to endothelial cell death.

To test if GB9 treatment decreased cell proliferation or migration, we examined ISV growth and the numbers of endothelial cells per ISV in the *Tg*(*kdrl:mCherry*; *fli1a:negfp^y7^*) embryos, where mCherry was expressed on endothelial ISV cells and GFP was expressed in the nucleus of endothelial cells. GB9 treatment showed slow or no growth at the middle of a somite and significantly reduced ISV cells compared to DMSO controls (Figure 4E–G, ISV *n* = 60 in GB9 treatment and *n* = 80 in controls, *p* < 0.0001). These results suggest that GB9 treatment disturbs ISV cell growth, likely by regulating the proliferation and/or migration of endothelial cells.

### 2.4. GB9 Treatment Reduces the Expression of Vascular Markers

**Figure 5:**
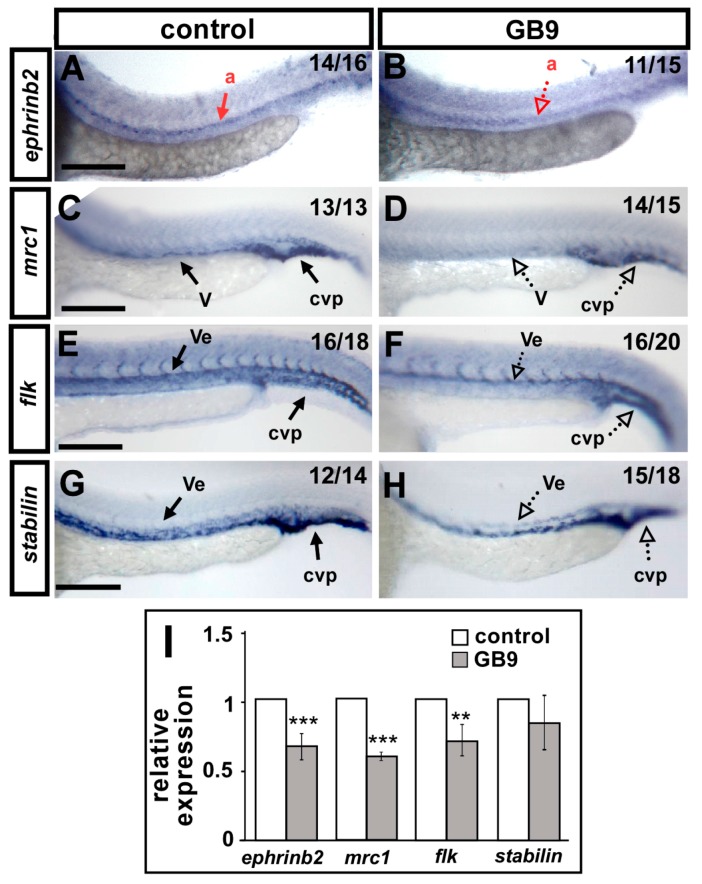
GB9 exposure reduces the expression of vascular markers. (**A**–**H**) Compared to wild-type controls (**A**,**C**,**E**,**G**), expression of the arterial marker *ephrinb2* (**B**), venous markers *mrc1* (**D**), pan-vascular marker *flk1* (**F**), and *stabilin* (**H**) were reduced in the trunk dorsal aorta (a), vein (v), vessels (ve), and caudal vein plexus (cvp) at 24 hpf in GB9 treatment embryos. (**I**) Quantification of the relative expression level by qPCR assay showed a ~40% and 30% reduced expression in vascular markers *ephrinb2* (0.65 ± 0.13), *mrc1* (0.58 ± 0.05), *flk* (0.7 ± 0.14) and *stabilin* (0.84 ± 0.2) in GB9 treatment embryos. Values on the top right indicate the number of embryos exhibiting phenotype per total number of embryos analyzed. *** refers to *p* < 0.0001 and ** refers to *p* < 0.001 by an unpaired Student’s *t*-test. Data are represented as means ± SD. Scale bars represent 200 μm in (**A**–**H**).

The vascular defects in ISV growth and CVP patterning suggested that GB9 treatment has impacts on vascular development and likely affects vascular identity. Thus, we examined the mRNA expression of vascular markers *ephrinb2*, *flk*, *mrc1*, and *stabilin* by whole-mount in situ hybridization at 24 hpf. We found that expression levels of the pan-vascular markers *stabilin* and *flk*, venous marker *mrc1*, and arterial marker *ephrinb2* were decreased in the trunk and CVP of GB9-treated embryos (dashed arrow in Figure 5B,D,F,H) compared to the wild-type. The decreased expression levels of vascular markers *ephrinb2*, *mrc1* and *flk* were quantified by qPCR and we found a 30–40% decreased expression in the GB9-treated embryos except *stabilin* (which showed to be slightly, but not significantly, decreased) (Figure 5I). The results suggest that GB9 treatment disrupts the expression of several vascular genes to inhibit vessel development. It is likely the early exposure of embryos beginning at 6 hpf may affect the specification of angioblasts, which could account for the decrease in expression of vascular markers and the defects of vasculature. Thus, we examined the expression of the early angioblast marker *scl* at the 12–14 somite stage (S) and showed no obvious difference between GB9-treated embryos and control (Figure S4).

### 2.5. GB9 Exposure Causes Vascular Defects Likely Mediated by Disruption of Redox Status

**Figure 6:**
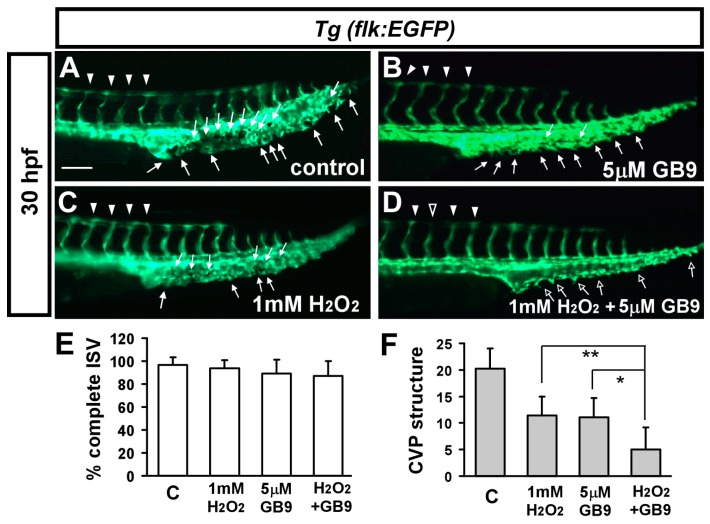
Enhanced effects of vascular defects while GB9 and H_2_O_2_ co-treatment. (**A**–**D**) Enhanced effects by combined lower dosage of GB9 treatment and lower dosage of H_2_O_2_ treatment embryos. At 30 hpf, in untreated control embryos (**A**), ISV have reached the DLAV (**arrowheads**) and the CVP with sprouting and/or loop (**arrows**) structures at the tail. Lower dosage of GB9 (5 μM) treatment embryos (**B**) or lower dosage of H_2_O_2_ (1 mM) treatment (**C**) showed mild or no effects in ISV growth and CVP development. Combination of GB9 treatment embryos and H_2_O_2_ treatment showed very mild vascular defects in ISV growth (**hollow arrowheads** in (**D**)) but strong effect in CVP formation (**less arrows** in (**D**)). (**E**,**F**) Quantification of percentage of completed ISV and CVP integrity showed an enhanced reduction in GB9 and H_2_O_2_ co-treated embryos (*n* = 11 for control, *n* = 10 for GB9 treatment, *n* = 11 for 1 mM H_2_O_2_ treatment and *n* = 11 for GB9, H_2_O_2_-cotreated embryos) at 30 hpf. ** refers to *p* < 0.001 and * refers to *p* < 0.01 by one-way ANOVA followed by Tukey’s post-test. Scale bars are 100 μm for (**A**–**D**).

H_2_O_2_ treatment induces oxidative stress and analysis of oxidative stress and has been applied to zebrafish embryos studies [35,37,38,39]. We hypothesized that the molecular mechanism by which GB9 impairs vascular development is mediated by an increase of oxidative stress. To test this hypothesis, we tested for potentially enhanced effects resulting from combining lower GB9 doses with lower H_2_O_2_ doses. If GB9 treatment increases oxidative stress, then co-treatment of H_2_O_2_ and GB9 will be more likely to cause vascular defects than GB treatment alone. At 30 hpf, in untreated control embryos, ISV reached the DLAV and the CVP formed loop structures at the tail (Figure 6A). Treatment with lower GB9 doses (Figure 6B) or lower H_2_O_2_ doses (Figure 6C) showed no significant effects in terms of ISV growth and only mild effects on CVP development (Figure 6E,F). Combined GB9 and H_2_O_2_ treatments showed severe vascular defects in CVP formation (Figure 6F), but not in ISV growth.

**Figure 7:**
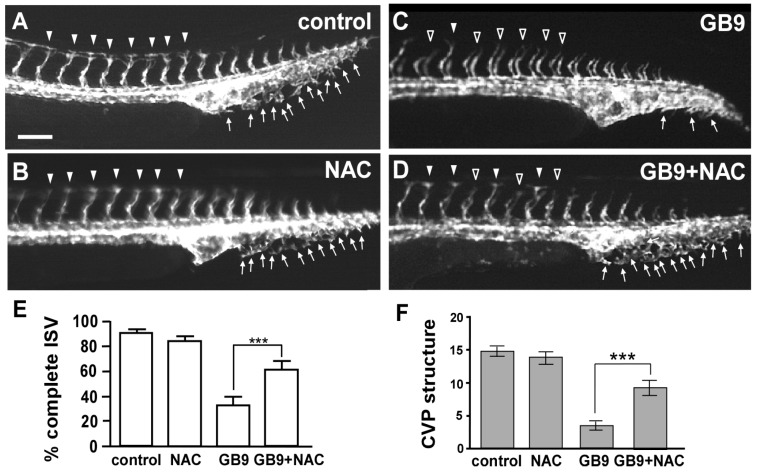
*N*-acetyl-cysteine (NAC) treatment rescues the vascular defects in GB9-treated embryos. (**A**,**B**) At 28 hpf, in untreated controls and 50 μM NAC-treated embryos, ISVs reached the DLAV at the embryo’s dorsal aspect (**arrowheads**) and endothelial sprouting at the CVP and some formed loop structures at the tail (**arrows**). (**C**) At the same stage, in GB9-treated embryos, ISVs showed slow growth mid-somite (**hollow arrowheads**) and less sprouting or loop formation at the CVP (**arrows**). (**D**) Exogenous 50 μM NAC exposure restores the vascular defects of ISV and CVP in GB9-treated embryos. (**E**) Percentages of completed ISV are respectively ~91 ± 2.2, 85 ± 2.9, 33 ± 5.9, and 62 ± 5.1 in controls, NAC treatment, GB9 exposure and rescued embryos. (**F**) Quantification of CVP structures respectively showed ~14.8 ± 0.8, 13.7 ± 0.9, 3.5 ± 0.8, and 9.2 ± 1.1 in controls, NAC treatment, GB9 exposure and rescued embryos. *** refers to *p* < 0.0001 by one-way ANOVA followed by Tukey’s post-test. Data represent means ± S.E. Scale bars are 100 μm for (**A**–**D**).

Moreover, incubating GB9-treated embryos with *N*-acetyl-cysteine (NAC), a known reactive oxygen species (ROS) inhibitor [40], alleviated the vascular defects of ISV and CVP (Figure 7A–F) as compared to control (Figure 7A). Completed ISV is found to be ~30% in GB9-treated embryos at 28 hpf 50 μM of NAC treatment in GB9-treated embryos restored the percentage of completed ISV to 62% and CVP structure to 60% of control (Figure 7E,F). These results show that coral-derived natural marine compound GB9 impairs vascular development likely mediated by the imbalance of redox homeostasis.

It has been shown that many antioxidant genes are induced, including *catalase*, *prdx1*, *prdx2*, *sod1*, and *sod2* while suffering oxidative stress by treatment with H_2_O_2_ [41]. Thus, we examined whether GB9 exposure causes the change of redox homeostasis. We showed GB9 treatment increases the expression of antioxidant genes *prdx1* and *sod1* significantly, and slightly induce, but not significantly, in *catalase* (*CAT*), *prdx2*, and *sod2* by qPCR analysis (Figure S5). These data imply that the GB9-treated embryos may suffer oxidative stress.

## 3. Discussion and Conclusions

Previous studies have found GB9 to respectively exhibit anti-neuroinflammatory and anti-nociceptive properties in IFN-γ-stimulated microglial cells and in neuropathic rats [10]. Thus, capnellene may develop as a new therapeutic agent for neuroinflammatory diseases. The present study analyzes the effects of GB9 treatment on zebrafish vascular function. We found a reduction of ISV cells and CVP endothelial cell sprouting and loop formation in GB9-treated embryos, indicating the impairment of cell proliferation and migration. Consistent with the vascular defects in GB9-treated embryos, we showed GB9 treatment resulted in the decreased expression of vascular markers. We further found an enhanced effect of GB9 and H_2_O_2_, and that NAC treatment rescued the vascular defects in GB9-treated embryos. These results suggest that GB9 exposure causes vascular defects mediated by an imbalance of redox environment.

In this study, we mainly focus on the effects of GB9 on vascular development. We also examined the impact of GB9-treatment on other tissue/organs development by assessing the gross developmental markers. Findings suggest that GB9 treatment does not affect the development of somites, blood or nerves in zebrafish embryos. Interestingly, the reduced expression of heart marker *cmlc2* and the reduced heart rate at 24–25 hpf suggest heart function could be disrupted. We currently do not know what mechanisms cause heart defects. Since this effect is in the early stages of heart development, it is possible that GB9 affects the differentiation of cardiomyocytes, heart tube formation and cardiac looping or builds atrial and ventricular chambers. In addition, endocardial cells and myocardial cells required for proper heart function could be affected by GB9 treatment. We also measured the body and eye size of zebrafish embryos at 48 hpf, and found no difference in body size, but a smaller eye diameter. The toxic effects of GB9 on eye development is interesting because we recently found that exposure to the environmental hormone pacobutrazol causes toxic effects on retinal photoreceptor cells growth, resulting in smaller eyes [42]. Future work should examine the potential toxicity of GB9 on the photoreceptor cells. In addition, retinoic acid is critical to eye development and its biosynthesis pathways could be disrupted to cause small eyes in GB9-treated embryos.

Marine resources have been intensively screened for novel potential drugs in our institute, and many marine compounds, such as GA2, Ya-s11, WA25, J10, GB9, GB10 and exc-B have been found to have anti-inflammatory properties [2,3,4,5]. However, not all resulted in vascular defects. Among those that do, WA25 inhibited angiogenesis by attenuating VEGF/VEGFR2 signaling [7] and GB9 impaired vascular development in this study. Other groups have reported anti-angiogenic effects among marine drugs, such as catunaregin which inhibits angiogenesis through the Akt and eNOS pathways. The present study shows that GB9 impairs vascular growth likely due to the redox imbalance, suggesting different mechanisms mediated by various marine drugs are attributed to angiogenesis. More importantly, our results provide useful information regarding the potential anti-angiogenesis effect of GB9 during treatment for inflammation or pain. In addition, the anti-angiogenic effect of GB9 may apply for the treatment of angiogenesis-related cancer or diabetes. Other marine drugs in our laboratory might induce angiogenesis and will be tested in the future.

The present study shows that GB9 treatment results in vascular defects during embryogenesis are mediated by an increase in oxidative stress, which is consistent with our recent findings that showed oxidative stress indeed interferes with vascularization [35]. However, the molecular mechanism behind this remains unclear, specifically what are the target genes of GB9 or what signals does GB9 regulate? We found that GB9 treatment downregulates the expression of *flk*/*VEGFR-2*, which has been reported to mediate most VEGF-A signals in endothelial cells, leading to cell proliferation, differentiation, migration, capillary formation, vascular permeability, and the angiogenic response. Therefore, the suppression of VEGF signaling pathways is considered a potential mechanism by which GB9 mediates angiogenesis inhibition. In addition, BMP signals have been shown to be important for CVP formation. Thus, future cellular-based research should seek to confirm the vascular effects of GB9, and to identify the interaction between VEGF or BMP signaling pathways and GB9 mediation of anti-angiogenesis. In addition, nitric oxide (NO) signaling has been reported to be critical to angiogenesis [43,44]. Whether GB9 treatment influenced NO homeostasis in endothelial cells is also intriguing. Thus, the high anti-angiogenic function and low toxicity of GB9 may have therapeutic potential for the treatment of angiogenesis-related diseases.

## 4. Materials and Methods

### 4.1. Zebrafish and Husbandry

Zebrafish (*Danio rerio*) wild-type AB stain and transgenic line: *Tg*(*kdrl*:*eGFP*)*^la116^*, *Tg*(*gata*:*dsRed*), *Tg*(*kdrl*:*mCherry*)*^ci5^*, and *Tg*(*fli1a*:*negfp*)*^y7^* from Taiwan Zebrafish Core Facility at Academia Sinica have been described [45,46,47,48] and were bred, raised, and maintained in a 28.5 °C fish room. The animal use protocol and experiment were approved by and conducted in accordance with the National Sun Yat-sen University Animal Care Committee (approval reference #10231).

### 4.2. Embryo Raising and Chemical Treatments

Zebrafish embryos were cultured in E3 media and dechorionated by incubation in 1 mg/mL Pronase (Sigma, St. Louis, MO, USA). Embryos pigmentation was inhibited by the addition of 0.003% 1-phenyl-2-thiouria (PTU) (Sigma) at 6 h post fertilization (hpf). Hydrogen peroxide (H_2_O_2_) (Sigma) treatment was made freshly. *N*-acetyl-cysteine (NAC), a common use for free radical scavengers [40], was obtained from Sigma. A 10 mg/mL stock solution was prepared in dd H_2_O and diluted in E3 fish medium to reach the desired concentrations 50 µM. GB9 was provided by Chang-Yih Duh’s laboratory. A 10 mg/mL stock solution was prepared in 0.3% dimethyl sulfoxide (DMSO) (Sigma) and diluted in E3 fish medium to reach the final desired concentrations. Control embryos were treated with an equivalent dose of DMSO alone. E3 media with chemical treatment was replaced daily.

### 4.3. RNA Preparation, cDNA Synthesis, and Real-Time Quantitative PCR (qPCR)

Total RNA from certain stages of embryos was extracted using the RNeasy kit (Qiagen, Valencia, CA, USA) according to the manufacturer’s instructions and with DNase I treatment to prevent genomic contamination. First-strand cDNA synthesis was performed with a mix of 1 μg total RNA, oligo-dT primer (Invitrogen, Philadelphia, PA, USA), and the RT-reverse transcriptase (Roche, Branford, CT, USA) according to the manufacturer’s instructions. Real-time quantitative PCR was performed using SYBR Green I Master (Roche) and the LightCycle 96 real-time PCR system (Roche). PCR primer sequences were designed using NCBI primer-BLAST and Supplemental Table S1 lists them. Relative cDNA expression were calculated using the LightCycle96 program and normalized to the expression level of β-actin based on the ΔΔC_t_ method. All experiments were performed as biological triplicates.

### 4.4. Riboprobe Generation and Whole-Mount In Situ Hybridization

Antisense riboprobes were synthesized using T7 polymerase with a DIG RNA labeling kit (Roche) according to the manufacturer’s instructions. The *ephrinb2*, *mrc1*, *flk*, and *stabilin* probes have been described [22,49,50]. The whole-mount in situ hybridization protocol was based on Thisse et al. [51]. Briefly, embryos were fixed with 4% paraformaldehyde and stored at −20 °C in methanol. After rehydration, the riboprobes were added to hybridization at 65 °C overnight. After washing and blocking, an AP-conjugated anti-Dig antibody (Roche) was added and reacted with the NBT/BCIP substrate (Roche). The embryos were embedded in 3% methylcellulose and photographed.

### 4.5. Imaging and Data Processing

Embryos were mounted in 3% methylcellulose (Sigma) or 1.5% low melting point agarose (Invitrogen) and image captured with a color digital AxioCam HRc camera (Carl Zeiss, Jena, Germany) or SPOT RT3 camera (Diagnostic Inc., Sterling heights, MI, USA). For the confocal images, embryos were embedded with 5% tricaine and images were collected on a Zeiss LSM700 or Nikon Eclipse 90i C1 confocal microscopes, and Z-stacked images (generally 30 slices with 5–10 μm between) were processed with ZEN software (Carl Zeiss) or ImageJ software (NIH, Bethesda, MD, USA).

### 4.6. Acridine Orange (AO) Staining

Dechorionated embryos were soaked in E3 fish medium containing 2 µg/mL acridine orange (Sigma) for 30 min. After washing with E3 water six times, embryos were mounted and photographed on a Lumar V12 microscope (Carl Zeiss) with a color digital AxioCam HRc camera (Carl Zeiss).

### 4.7. Terminal Deoxynucleotidyl Transferase dUTP Nick End Labeling (TUNEL) Assay

A TUNEL analysis was performed using an in situ cell death detection kit (Roche) according to the manufacturer’s instructions and our previous study [52]. Embryos were fixed and stored in methanol. After rehydration, then permeabilization with proteinase K (Roche), embryos were re-fixed in 4% PFA for 15 min, then treated with 3% H_2_O_2_ to eliminate endogenous peroxidase, followed by incubation with 45 µL of TUNEL (TdT-mediated dUTP-X nick end labeling) label solution plus 5µL of TUNEL enzyme for 3 h at 37 °C in the dark. Embryos were then washed with PBT, blocked with 5% Serum (Thermo Scientific, South Logan, UT, USA) in PBT, incubated with peroxidase (POD) conjugated anti-fluorescein antibody (Roche) overnight in the dark, then washed four times in PBT, and visualized using DAB substrate.

### 4.8. Statistical Analysis

Statistical analysis was performed with Student’s *t*-test or one-way analysis of variance (ANOVA) followed by Tukey’s post-test using GraphPad PRISM version 5.0 (GraphPad, San Diego, CA, USA). A *p* < 0.05 indicates statistically significance.

## Supplementary Materials

Supplementary materials can be found at www.mdpi.com/1422-0067/18/8/1696/s1.
