# Supplementary material for: Coral-Derived Natural Marine Compound GB9 Impairs Vascular Development in Zebrafish
Source: Int J Mol Sci. 2017 Aug 3;18(8):1696. doi: 10.3390/ijms18081696 (PMC5578086; doi:10.3390/ijms18081696)
Supplement: Supplementary file 1 [file ijms-18-01696-s001.pdf]

Supplementary Materials:

Coral-Derived Natural Marine Compound GB9 Impairs Vascular Development in Zebrafish

Yi-Chun Song[1†], Bao-Jueng Wu [2,3†], Chien-Chih Chiu[1,4,5†], Chun-Lin Chen[1], Jun-Qing Zhou[1], Shuo-Rong Liang[1], Chang-Yih Duh[2], Ping-Jyun Sung[6], Zhi-Hong Wen[2,7] and Chang-Yi Wu[1,4,7*]

Table S1. Quantitative PCR (qPCR) primer sequences used in this study.

qPCR Primers	Sequence
*β-actin*_qf	5′-CTCTTCCAGCCTTCCTTCCT-3′
*β-actin*_qr	5′-CTTCTGCATACGGTCAGCAA-3′
*flk1*_qf	5′-ACTTTGAGTGGGAGTTTCATAAGGA-3′
*flk1*_qr	5′-TTGGACCGGTGTGGTGCTA-3′
*mrc1*_qf	5′-CTAGCAAGCCTGAAGGTGCC-3′
*mrc1*_qr	5′-TGAGAGGCTGGGTAGTTGGG-3′
*ephrinb2*_qf	5′-CTGGAACACCACGAACACC-3′
*ephrinb2*_qr	5′-CACACGTGGGCAAACTATGT-3′
*stabilin*_qf	5′- GGGCTTCCAATACCAACTGG -3′
*stabilin*_qr	5′- CCTGGTTGCACAGACAGACC -3′
*sod1*_qf	5′-GTTTCCACGTCCATGCTTTT-3′
*sod1*_qr	5′-CGGTCACATTACCCAGGTCT-3′
*sod2*_qf	5′-AGCGTGACTTTGGCTCATTT-3′
*sod2*_qr	5′-TCTTCCGCTCTCCTTTTCAA-3′
*cat*_qf	5′-CGCTTCTGTTTCCGTCTTTC-3′
*cat*_qr	5′-GGAATCCCTCGATCACTGAA-3′
*prdx1*_qf	5′-ACACATTGGGAAACCTGCTC-3′
*prdx1*_qr	5′- GCACCACGTACTTCCCTTTG-3′
*prdx2*_qf	5′-GTCAGCCTGCTCCTCAGTTC-3′
*prdx2*_qr	5′-TGGGACACACAAAGGTGAAA-3′

Supplementary Figures

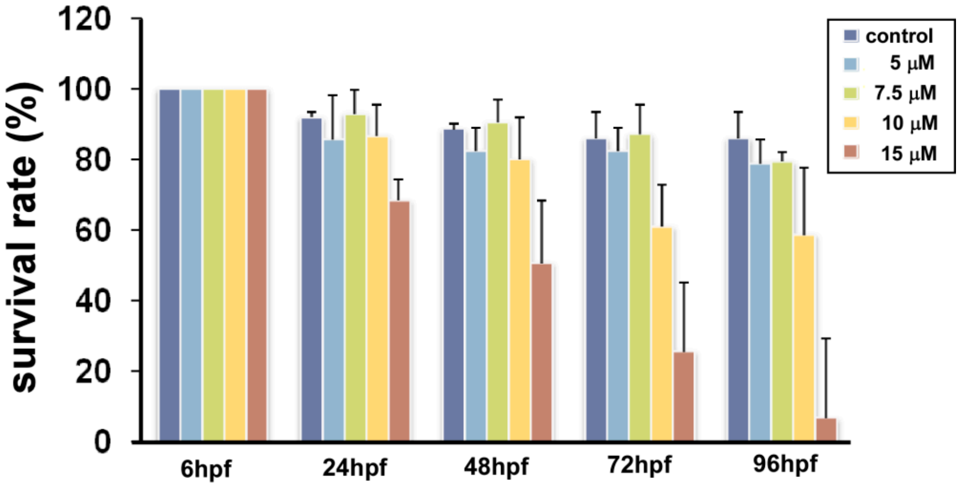

Figure S1. Survival rate of GB9-treated embryos. Wild type zebrafish embryos were treated with various concentrations of GB9 (0, 5, 7.5, 10, and 15 μM) at 6 hpf, with survival rates recorded at 24, 48, 72 and 96 hpf. The survival ratio at 6 hpf is set as 100%. These experiments were conducted in triplicate from 3 independent sets. All data were shown as the mean ± S.D.

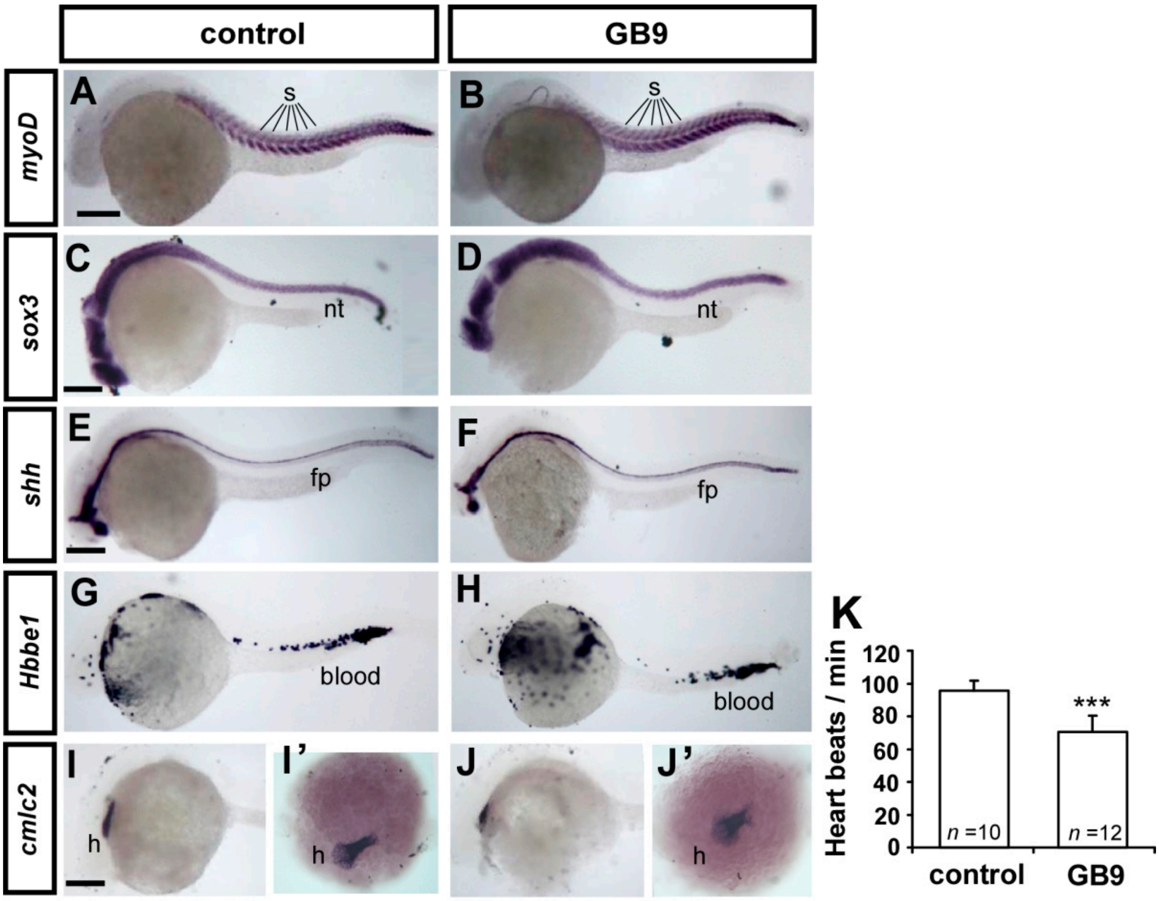

Figure S2. Gross developmental process in GB9-treared embryos. (A-J, I′-J′) Embryos treated with 10μM GB9 did not alter the expression pattern of somite, neural systems, and blood by examining the expression of *myoD* (somite marker, s, **A, B**), *sox3* (neural tube marker, nt, **C, D**), *shh* (floor plate marker, fp, **E, F**), and *Hbbe1* (blood marker, **G, H**). However, GB9-treated embryos showed slightly reduced and smaller expression area of heart marker *cmlc2*. (h; **I-J** are lateral view and **I′-J′** are ventral view) **(K)** Quantification of zebrafish heart beats per minute in 0.2% DMSO control (n=10) and 10 μM GB9 treated embryos (n=12). The mean heart rate determined by direct visual examination of ventricle beating in control was 95.8 ± 6.0 beats per minute, 70.4 ± 9.8 beats per minute in GB9-treated embryos. *** refers to $p < 0.0001$ by an unpaired student's t-test. The scale bars represent 250 μm in **A-J.**

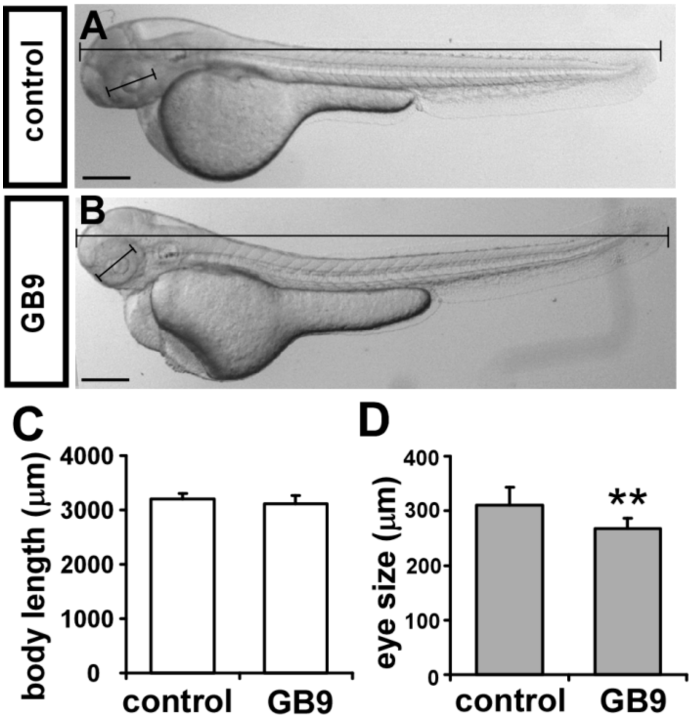

Figure S3. The effects of GB9 on the size of the body and eye in zebrafish embryos. GB9 exposure did not change the body length **(A, B, C)**, but reduces the eye size **(A, B, D)** in zebrafish embryos compared to 0.3% DMSO control. The body length is 3204.9 ± 96.3 μm in control and 3111.9 ± 148.8 μm in GB9-treated embryos (p=0.168) as measured from head to the end of tail fin. The eye size is 310.6 ± 32.5 μm in control and 268.1 ± 17.8μm in GB9-treated embryos (p<0.005) as determined the diameter of eye. The images are the representative pictures from two independent experiments and data are shown as the mean ± S.D. ** refers to p < 0.005 by an unpaired student's t-test. The scale bars represent 250 μm in **A-B.**

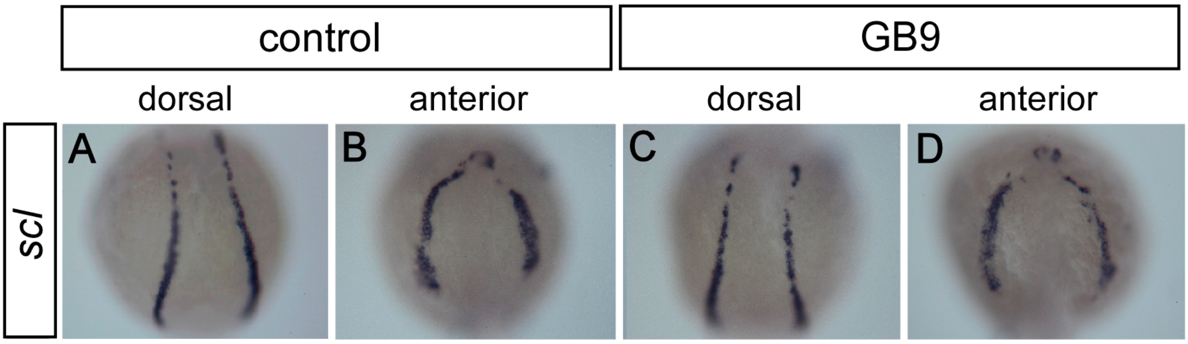

Figure S4. GB9 treatment did not alter the expression of the angioblast marker *scl*. (A-D) Expression of angioblast marker *scl* in 0.3% DMSO control **(A, B)** and GB9-treated embryos **(C, D)** at 14 somite stages (S) as seen in dorsal or anterior views.

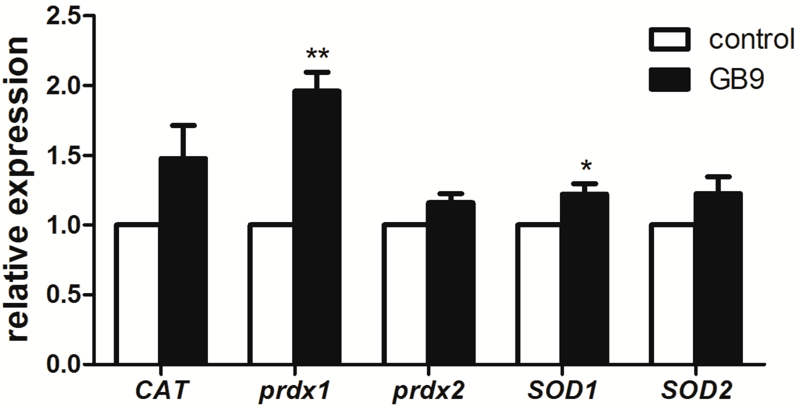

Figure S5. Remodeling the expression of antioxidant genes in GB9-treated embryos. GB9 exposure increases the expression of antioxidant genes *prdx1* (2.0 ± 0.23) and *sod1* (1.22 ± 0.13) significantly, but no difference in *catalase (CAT)* (1.5 ± 0.41), *prdx2* (1.16 ± 0.12) and *sod2* (1.22 ± 0.21) by qPCR analysis. *β-actin* serves as a loading control. (** refers to $p < 0.005$ and * refers to $p < 0.05$ by an unpaired Student's t-test)
